# Evaluation of efficacy and safety of lascufloxacin for nursing and healthcare associated pneumonia: single-arm, open-label clinical trial: A study protocol

**DOI:** 10.1097/MD.0000000000033092

**Published:** 2023-02-22

**Authors:** Naoki Hosogaya, Takahiro Takazono, Kenji Ota, Rieko Kiya, Yumi Shirai, Rina Kawasaki, Hiroshi Yano, Shinpei Morimoto, Rumiko Nakao, Yumiko Kanamaru, Yukari Yoshino, Yasuyuki Ishikawa, Chizu Fukushima, Hiroshi Yamamoto, Koichi Izumikawa, Katsunori Yanagihara, Hiroshi Mukae

**Affiliations:** a Clinical Research Center, Nagasaki University Hospital, Nagasaki, Japan; b Department of Respiratory Medicine, Nagasaki University Hospital, Nagasaki, Japan; c Department of Infectious Diseases, Nagasaki University Graduate School of Biomedical Sciences, Nagasaki, Japan; d Department of Laboratory Medicine, Nagasaki University Hospital, Nagasaki, Japan; e Medical affairs, KYORIN Pharmaceutical Co.,Ltd., Tokyo, Japan; f Department of Respiratory Medicine, Nagasaki University Graduate School of Biomedical Sciences, Nagasaki, Japan.

**Keywords:** lascufloxacin, LSFX, NHCAP, nursing and healthcare associated pneumonia

## Abstract

**Background:**

Lascufloxacin hydrochloride (LSFX) is a quinolone antibiotic that inhibits DNA gyrase and topoisomerase IV of bacteria, it is anticipated to minimize antibiotic resistance in bacteria. It exhibits antibacterial activity against a relatively wide range of bacterial species, including anaerobic bacteria, and its efficacy and safety against community-acquired pneumonia have been shown; however, its efficacy and safety against nursing and healthcare associated pneumonia (NHCAP) have not been verified.

**Methods/Design:**

Here, a single-arm, open-label, uncontrolled study was conducted in which LSFX was administered to patients with NHCAP at 24 facilities. The research subjects (77 cases) were orally administered 75 mg of LSFX once a day for 7 days. The primary endpoint was the clinical efficacy at the time of test of cure (TOC) (TOC; 5–10 days after the end of LSFX administration), while the secondary endpoints were the efficacy at the time of end of treatment, early clinical efficacy, microbiological efficacy at the time of TOC and end of treatment, and safety evaluation of LSFX.

**Discussion:**

NHCAP is a common pneumonia in clinical settings and a notable pneumonia whose mortality is high compared to community-acquired pneumonia. The present study showed the efficacy and safety of LSFX against NHCAP, which could lead to a larger number of therapeutic options for NHCAP.

## 1. Introduction

Pneumonia is a major infection that was the 4th leading cause of death in Japan in 2011.^[1]^ In Japan, pneumonia is classified into community-acquired pneumonia (CAP), nursing and healthcare associated pneumonia (NHCAP), and hospital-acquired pneumonia based on the site of onset and risk of resistant bacteria. Mortality is different for each type: 6.3%, 15.5%, and 30.4%, respectively, thus, appropriate evaluation and therapeutic options are needed.^[2]^ For CAP and NHCAP, severity is evaluated based on the occurrence of sepsis and with the A-DROP system, and an antibacterial drug is selected for treatment. However, in the case of NHCAP, the risk of resistant bacteria and severity are also considered to select an escalation or de-escalation treatment.

Lascufloxacin hydrochloride (LSFX) is a quinolone antibiotic that exhibits antibacterial activity against a relatively wide range of bacterial species, including anaerobic bacteria, by inhibiting DNA gyrase and topoisomerase IV of bacteria. While the existing quinolone antibiotics strongly inhibit either DNA gyrase or topoisomerase IV, LSFX inhibits both to a similar degree. Thus, it is anticipated to limit antibiotic resistance in bacteria.^[3–6]^

In a Japanese Phase III trial, noninferiority of LSFX against levofloxacin in clinical efficacy at the time of test of cure (TOC) against CAP was verified and its safety was shown to be acceptable; however, there are no data available on the efficacy and safety against NHCAP.^[7]^ Many cases of NHCAP are caused by aspiration and often lead to repetitive pneumonia; thus, LSFX, a drug that is unlikely to lead to resistant bacteria, and could become a useful treatment option.

Presently, we are conducting an open-label, uncontrolled study in which LSFX is administered to patients with NHCAP. The study aims to confirm the rate of cure and adverse events 7 days after the end of treatment (EOT) with LSFX as well as search for the rate of cure and safety profile of LSFX against NHCAP. Herein, we describe the final protocol (version 4.0; April 1, 2022).

## 2. Methods/design

### 2.1. Study design

This clinical trial was designed in accordance with the Standard Protocol Items: Recommendations for Interventional Trials and Consolidated Standards of Reporting Trials 2010 guidelines.^[8,9]^ This is an investigator-initiated, multicenter, open-label, and interventional clinical trial to investigate the efficacy and safety of lascufloxacin for NHCAP.

### 2.2. Study approvals

This clinical trial was approved by the certified review board (CRB) of Nagasaki University (CRB approval no.: CRB20-023) and is registered with the Japan Registry of Clinical Trials (trial no.: jRCTs071200066). This study will be conducted at the following 24 centers: Nagasaki University Hospital; Japanese Red Cross Nagasaki Genbaku Hospital; Saiseikai Nagasaki Hospital; Japanese Red Cross Nagasaki Genbaku Isahaya Hospital; Japan Community Healthcare Organization Isahaya General Hospital; Aino Memorial Hospital; Kouseikai Hospital; Nagasaki Harbor Medical Center; Sasebo City General Hospital; Sasebo Chuo Hospital; Ureshino Medical Center; Senju Hospital; Izumikawa Hospital; Tobata Kyoritsu Hospital; Kurate Hospital; Tobata General Hospital; Kirigaoka Tsuda Hospital; Kitakyushu General Hospital; Hospital of the University of Occupational and Environmental Health, Japan; Wakamatsu Hospital of the University of Occupational and Environmental Health, Japan; Nagasaki Goto Chuoh Hospital; Nagasaki Memorial Hospital; Shimabara Hospital; and Nagasaki Kidney Hospital. This trial will be performed in accordance with the principles of the Declaration of Helsinki, Japan Clinical Trials Act (Act No. 16 of April 14, 2017), Japan Act on the Protection of Personal Information and related regulatory notifications, and the present study protocol.^[10,11]^

### 2.3. Participant recruitment

Participants will be recruited from the aforementioned 24 hospitals. All eligible patients will be enrolled according to the inclusion and exclusion criteria. Participants or legally acceptable representatives will be provided with information regarding the study by their respective respiratory specialists and requested to sign an informed consent form if they are willing to participate.

### 2.4. Inclusion criteria

Patients should meet the following criteria to be enrolled:

(1) Patients who are older than 20 at the time of providing informed consent.
(2) Patients who present acute manifestation of obvious infiltrates in a chest X-ray or high-resolution computed tomography image obtained within 48 hours of administration of the study drug.
(3) Patients who meet both 1) and 2):
  1) Patients who have at least one of the following symptoms:
    a. Cough.
    b. Purulent sputum or sputum with increased purulence.
    c. Abnormal findings in auscultation or percussion (moist rales, dullness to percussion, decreased breathing sounds, etc).
    d. Worsening of either or both dyspnea and tachypnea.
    e. Fever: ≥ 37°C (axillary temperature).
  2) Patients who have at least one of the following:
    a. Increased C-reactive protein (CRP) level.
    b. Increased white blood cell count (> 10,000/ mm^3^), or stab cells > 15%.
    c. Hypoxemia (PaO_2_ < 60 Torr or SpO_2_ < 90%).
(4) Patients who are classified as mild or moderate by the A-DROP scoring system.^[12]^
(5) Patients who meet at least one of the following:
  1) Admittance to a long-term care or nursing home.
  2) Discharge from hospital in the preceding 90 days.
  3) Elderly or physically disabled people who require care.
  4) Outpatients who regularly receive infusion therapy (including dialysis, antibiotics, anticancer agents, immunosuppressant drugs, etc.) or hemodialysis.
(6) Patients who are able to take drugs orally.
(7) Written informed consent has been provided by the patient or legally acceptable representative to participate in the study.

### 2.5. Exclusion criteria

The exclusion criteria are as follows:

(1) Patients with a previous history of hypersensitivity or serious adverse reaction to quinolones.
(2) Patients with complications or a past history of a convulsive disorder, such as epilepsy.
(3) Female patients who are pregnant or may be pregnant.
(4) Patients with severe dysfunction in the liver or heart.
(5) Patients with severe or progressive underlying disease or complication (patients with diabetes with HbA1c > 8.0% regardless of treatment, patients with advanced cancer whose prognosis cannot be expected during the observation period, patients with advanced cancer requiring surgery, etc).
(6) White blood cell count ≤ 2000/ mm^3^ at the medical facility.
(7) Patients with a severe infection requiring treatment with intravenous antibacterials or mechanical ventilation.
(8) Patients with bronchial obstruction or a past history of obstructive pneumonia (but not excluding patients with chronic obstructive pulmonary disease).
(9) Patients with primary lung cancer or lung metastasis of a malignant tumor that highly affects the clinical evaluation of pneumonia treatment.
(10) Pneumocystis pneumonia (including suspected cases) or active pulmonary tuberculosis (including suspected cases). Patients with eosinophilic pneumonia, interstitial pneumonia, or cystic fibrosis that highly affects the clinical evaluation of pneumonia treatment.
(11) Patients who received a systemic antibacterial within 7 days prior to the administration of the study drug (including long-term, low dose macrolide therapy).
(12) Respiratory infections caused by pathogens (acid-fast bacilli, fungi, viruses, etc) for which the study drug is not expected to be effective.
(13) Patients with active hepatitis B or C virus infection, or HIV infection.
(14) Patients who are judged to be unsuitable for participation in this trial by the principal or subinvestigator.

### 2.6. Study protocol

A principal investigator or a subinvestigator of participating medical institutions provides a sufficient explanation to patients or proxies using the consent form and documents approved by the Review Board of Certified Clinical Trials before patients participate in the study. After confirming that the patients or proxies sufficiently understand the details of the study, consent to participate is obtained in writing. The principal investigator or subinvestigator of participating medical institutions confirms that the patients meet all of the inclusion criteria and do not meet any of the exclusion criteria, and input all necessary items into an electronic data capture (EDC) system. When determined to be eligible, an ID code is issued by the EDC system as the case registration number. Patients with NHCAP are orally administered 75 mg of LSFX (Lasvic^®^ tablet) once daily for 7 days. The duration of administration is 7 consecutive days. If the attending physician determines that continued administration is necessary, administration for up to 10 days is possible. Tests and observations are undertaken on day 3 of administration, at the end of treatment, and 1 week after the end of administration (5–10 days) (Table 1).

**Table 1:**
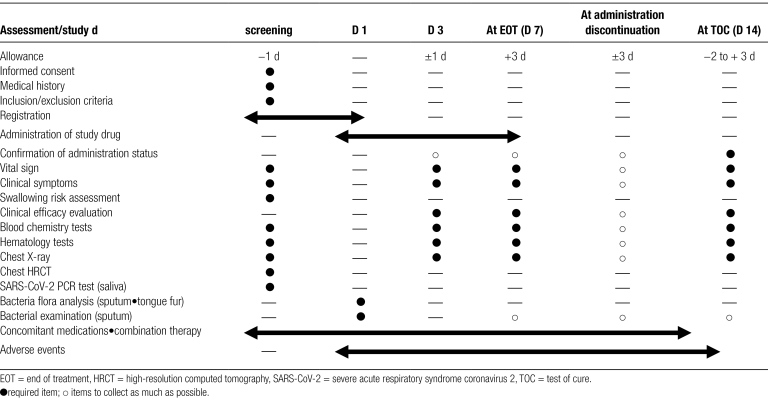
Treatment schedule and outcome measures.

In this study, there is no drug or therapy that must be used in combination, but the use of the following drugs during the study period requires caution: formulations that contain aluminum, magnesium, calcium, iron, or zinc; phenylacetic acid- and propionic acid-nonsteroidal antiinflammatory analgesics; rifampicin, phenytoin, or carbamazepine; theophylline or aminophylline hydrate; class IA or III antiarrhythmic drugs; and adrenal corticosteroids (oral agents or injections).

Criteria to discontinue the study are as follows:

• If a patient or their proxy requests to withdraw their consent.
• A principal investigator or subinvestigator determines that it is difficult to continue with the study due to illness, etc.
• A principal investigator or subinvestigator determines that it is difficult to continue with the study due to adverse events.
• If the patient becomes pregnant.
• If the patient tests positive for SARS-CoV-2.
• A principal investigator or subinvestigator determines that it is difficult to continue with the study for any other reason.

### 2.7. Adverse events

In the case of adverse events, a principal investigator or subinvestigator of participating medical institutions must promptly take appropriate treatment measures for the patients (including providing an explanation), discontinue or pause the clinical study, and take any other necessary measures. If the adverse events are still present at the time of final observation, the principal investigator or subinvestigator of the participating medical institutions should observe the course until the subjects recover to the state prior to the onset of the adverse events or until they reach a clinically stable state. The principal investigator or subinvestigator should evaluate the adverse events and input them into the EDC system. Severe adverse events are classified into death, illnesses that could lead to death, illnesses that require hospitalization at a medical institution for treatment or extended hospitalization, disabilities, illnesses that could lead to disabilities, or diseases or abnormalities in later generations.

An independent Data and Safety Monitoring Committee will not be established. If participating in this study causes health damage to the patients, the principal investigator or subinvestigator will provide appropriate treatment and other necessary measures. If there is compensation liability for health damages caused by this study, and if patients die or suffer permanent disabilities, said patients will be compensated through clinical research compensation liability insurance.

### 2.8. Outcome measures

#### 2.8.1. Early efficacy evaluation 3 days after the first dose of the study drug.

The efficacy evaluation 3 days after the first dose of the study drug will result in the following categorizations: effective (in addition to improvements in 1 or more clinical symptoms there will be improvements to pneumonia images or inflammatory findings; WBC < 9000/ mm^3^ or a decrease from the highest CRP level), no effect, or indeterminable (lack of evaluation information or evaluation items worsening due to causes other than pneumonia).

#### 2.8.2. Efficacy evaluation at the time of EOT.

The efficacy evaluation at the time of EOT will result in the following categorizations: effective (in addition to improvements in 1 or more clinical symptoms accompanied by resolution of fever, there will be improvements to pneumonia images or inflammatory findings; WBC < 9000/ mm^3^ or a decrease from the highest CRP level), ineffective, or indeterminable (lack of evaluation information or evaluation items worsening due to causes other than pneumonia).

#### 2.8.3. Efficacy evaluation at the time of TOC.

TOC will be determined 5 to 10 days after the last dose of the study drug, with subjects classified as being cured, not cured, or indeterminable (lack of evaluation information, evaluation items worsening due to causes other than pneumonia, or clinical symptoms or inflammatory findings improved but the antibacterial drug was prescribed for reasons other than NHCAP before the time of TOC). Cured satisfies all the following criteria: treatment determined to be effective at the time of EOT; or an additional prescription of an antibacterial drug is not necessary after the EOT to the TOC; body temperature, WBC count, CRP level did not deteriorate after EOT, and resulting in an additional prescription of an antibacterial drug not being required; improved symptoms compared to the EOT; and improved chest X-ray compared to the EOT.

#### 2.8.4. Microbiological efficacy evaluation.

The microbiological efficacy is evaluated for each causative bacterium and subject as disappeared, persistent, or indeterminable using the following criteria:

Disappeared, symptoms improved and meet the following:

• Disappeared: Causative bacteria not detected in specimens.
• Presumed disappearance: Specimens cannot be obtained.
• Colonization: The same causative bacterium is submitted.

Persistent, symptoms do not improve and meet the following:

• Persistent: Causative bacterium is detected in specimens.
• Presumed persistence: Culturing of the specimen is difficult or is not performed.
• Relapse: The same causative bacterium is detected.
• Microbial substitution: The causative bacterium has disappeared but a new causative microorganism is detected (limited to the evaluation of subjects only).
• Coinfection: The causative bacterium persists and a new microorganism is detected (limited to the evaluation of subjects only).

Indeterminable, test was not performed.

### 2.9. Study endpoints

#### 2.9.1. Primary endpoint.

The primary endpoint is the efficacy evaluation at the time of TOC (5–10 days after the EOT for pneumonia).

#### 2.9.2. Secondary endpoints.

The secondary endpoints are as follows: Clinical efficacy at the time of EOT; Early clinical efficacy 3 days after the first administration; Microbiological efficacy at the time of TOC (each causative bacteria, each subject); Microbiological efficacy at the time of EOT (each causative bacteria, each subject).

#### 2.9.3. Safety endpoints.

All adverse events that occur during the trial will be recorded.

#### 2.9.4. Exploratory.

The following stratified analyses will be performed for the primary and secondary endpoints: risk of antimicrobial resistance (no or yes),^[2]^ risk of aspiration by (standardized swallowing assessment [SSA]; no or yes),^[13]^ and first to third occupied bacterial species by comprehensive bacterial flora analysis (clone library method).^[14]^

### 2.10. Data collection and management

In this study, data of each patients will be collected by web-based EDC system that electronically prepares case reports. A principal investigator or subinvestigator of the participating medical institutions will input the data needed for the study protocol according to Table 1 to the electric case report in the EDC system. The principal investigator of the participating medical institutions will electronically sign the case reports and ensure that the input data are complete and accurate.

The principal investigator or subinvestigator of the participating medical institutions will confirm that patients meet all the inclusion criteria and do not meet any of the exclusion criteria and input all necessary items into the EDC system. When determined to be eligible, the case registration number issued by the EDC system will be used. The case registration number consists of numbers and symbols unrelated to the information that could identify specific individuals, such as initials and file IDs. When preparing documents related to this study, such as case reports, subjects will be anonymized by the use of case registration number. The principal investigator will prepare a table that shows the patient’s name, number, and so on, so that they can be identified if necessary. This table will be strictly stored and managed so that is cannot be externally leaked. Those involved with this study will make utmost efforts to protect personal information and privacy and are not permitted to share personal information acquired through this study externally without valid reason and permission.

The sponsor-investigator will perform monitoring to review compliance of protocol, each standard operating procedure, and various ministrial ordinances, and reliability of the data. In this study, with the aim of ensuring reliability of this trial, external audit will be conducted. Monitoring and audit will be conducted according to standard operating procedure of monitoring/ audit.

### 2.11. Sample size considerations

The sample size was determined to be the minimum value, with which the lower limit of the 95% confidence interval (CI) (Clopper–Pearson method) of the binomial probability is lower than 0.10, when the point estimate of the probability is 0.875. The point estimate was presumed as it is the same with the probability of NHCAP cure with a 7 days administration of moxifloxacin in an existing report.^[15]^ Further, assuming that 0.30 of incorporated cases would be evaluated as “indeterminable,” the target case number was set to 77.

### 2.12. Statistical analysis

The target population in safety analyses (SAS) was defines as patients registered as study participants and administered the study drug at least once during the study period. The target population of the full analysis set (FAS) was defined as a subset of SAS consist of patients who are able to be evaluated whether “cured” or “not cured” at TOC. The modified FAS was defined as an extension of the FAS that includes patients who are able to be evaluated whether “cured” or “not cured” at the TOC or at the withdrawal from the protocol treatment. The target population in the per protocol set analysis was defined as patients with adherence of equal or higher than 80% to the protocol treatment (doses of the study drug).

The objective of the primary analysis is to obtain the lower limit of the 95% CI of the binomial probability of frequency of “cured.” The Clopper–Pearson method was planned to be used here. The FAS was planned as the target population.

A sensitivity analysis for the primary analysis is planned to evaluate the upward bias in the estimation of the probability of the treatment success, that will be introduced by the exclusion of cases who are withdrawn from the protocol treatment before TOC evaluation, obtained from the primary analysis. Aiming this, the modified FAS was set as the target population of the sensitivity analysis and the 95% CI of the binomial probability was planned to be calculated via the Clopper–Pearson method.

The planned analyses on the secondary endpoints are same with the primary analysis; the 95% CI of the binomial probability but the target population, that was set on the FAS.

Associations of the patients background and the primary/ secondary endpoints were planned to be analyzed exploratorily. The patients background was defined as the results from evaluations on respective patients at the visits for the first administration, or before the date, during the patient’s study period.

Planned subgroup analyses are the same procedure used in the primary analysis, after stratification of the patients with the following respective factors: risk of resistant bacteria (yes/no), aspiration risk assessed by standardized swallowing assessment (yes/no), and the first to the third most dominant bacterial species (presence/absence of bacteria with specific physiology, such as anaerobic bacteria, or types of bacteria) analyzed by a clone library method using amplified fragments of the 16S ribosomal RNA gene with universal primers in addition to conventional cultivation methods. The p-values obtained through the subgroup analyses will not be used to show the type I error rates for rejections of the respective null hypotheses.

## 3. Discussion

The efficacy and safety of LSFX for CAP have been shown in clinical trials. NHCAP is pneumonia with a higher risk of resistant bacteria and death compared to CAP. The described study will evaluate the efficacy and safety of LSFX for NHCAP.

LSFX is a quinolone antibiotic that exhibits antibacterial activity against a relatively wide range of bacterial species, including anaerobic bacteria, by inhibiting DNA gyrase and topoisomerase IV.^[3,4]^ Its efficacy and safety against respiratory and otorhinolaryngological infections have been shown in a phase III trial, and it received pharmaceutical approval in September 2019. While the existing quinolone antibiotics strongly inhibit either DNA gyrase or topoisomerase IV, LSFX inhibits both to a similar degree. Thus, it is anticipated to minimize resistant bacteria.^[3,5,6]^ Aspiration is common in NHCAP and pneumonia often recurs, thus, LSFX with a limited risk of resistant bacteria could be a useful option.

LSFX is an oral antibacterial drug and is intended for patients with NHCAP with mild-to-moderate symptoms that could be treated with oral formulations. Though injectable monotherapy is recommended for NHCAP with a risk of resistant bacteria, in actual clinical settings, NHCAP cases that can be treated in an outpatient setting are treated with respiratory quinolones. A risk of resistant bacteria is not necessarily a prognostic factor,^[4]^ and the described study uses a system that allows prompt change in treatment upon appropriately evaluating the condition on Day 3 and 7 of treatment. As such, if a principal investigator or subinvestigator determines it to be appropriate, patients with a risk of resistant bacteria are also allowed to participate.

Based on the evaluation method for NHCAP, according to respiratory infections in the guidelines related to the clinical evaluation methods for antibacterial drugs,^[15]^ the primary endpoint will be set as the TOC. This is the same as the efficacy evaluation for CAP. An early-stage efficacy evaluation will also be set for the secondary endpoints, but these are items recommended as primary endpoints in the Guidance Document for Community-Acquired Bacterial Pneumonia: Developing Drugs for Treatment published by the FDA.^[16,17]^ Thus, evaluation items that could be compared with pneumonia treatment results from overseas will also be included.

The study will evaluate the first to third most dominant bacterial species through exhaustive microbiome analysis (clone library method) of the tongue coating and sputum at the time of pneumonia diagnosis. Oral bacteria include many anaerobic bacteria and the detection of bacteria is extremely difficult in a normal culture test. However, the exhaustive microbiome analysis does not require culture, and exhaustively detects the 16s rRNA gene; thus, it is an especially useful detection method for bacterial species when culturing is difficult.^[18]^ Since many cases of NHCAP are aspiration pneumonia, we assume that by comparing the microbiome in the tongue coating and sputum, the relationship between NHCAP and oral bacteria can be evaluated.

In the study, the efficacy and safety of LSFX against NHCAP, will be presented with the hope of increasing the therapeutic options for NHCAP. The study will be a significant contribution in establishing an appropriate therapeutic system.

## Acknowledgments

We would like to thank Editage (www.editage.com) for English language editing. We would also like to thank the 23 cooperating facilities and their respective principal investigators: Koji Hashiguchi (Japanese Red Cross Nagasaki Genbaku Hospital), Yoji Futsuki (Saiseikai Nagasaki Hospital), Yosuke Nagayoshi (Japanese Red Cross Nagasaki Genbaku Isahaya Hospital), Tomo Mihara (Japan Community Healthcare Organization Isahaya General Hospital), Yuichi Inoue (Aino Memorial Hospital), Yosuke Harada (Kouseikai Hospital), Toyomitsu Sawai (Nagasaki Harbor Medical Center), Yuichi Fukuda (Sasebo City General Hospital), Tsutomu Kobayashi (Sasebo Chuo Hospital), Eisuke Sasaki (Ureshino Medical Center), Hikaru Tanaka (Senju hospital), Ryosuke Morio (Izumikawa Hospital), Hidenori Ohira (Tobata Kyoritsu Hospital), Tetsuya Hanaka (Kurate Hospital), Shingo Noguchi (Tobata General Hospital), Tetsuya Kawano (Kirigaoka Tsuda Hospital), Tsuyoshi Orihashi (Kitakyushu General Hospital), Kazuhiro Yatera (Hospital of the University of Occupational and Environmental Health, Japan), Chiharu Yoshii (Wakamatsu Hospital of the University of Occupational and Environmental Health, Japan), Takayuki Suyama (Nagasaki Goto Chuoh Hospital), Toru Morikawa (Nagasaki Memorial Hospital), Takuto Miyamura (Shimabara Hospital), and Satoshi Funakoshi (Nagasaki Kidney Hospital).

## Author contributions

**Conceptualization:** Naoki Hosogaya, Takahiro Takazono, Kenji Ota, Rieko Kiya, Yumi Shirai, Rina Kawasaki, Hiroshi Yano, Shinpei Morimoto, Rumiko Nakao, Yumiko Kanamaru, Yukari Yoshino, Yasuyuki Ishikawa, Chizu Fukushima, Hiroshi Yamamoto, Koichi Izumikawa, Katsunori Yanagihara, Hiroshi Mukae.

**Data curation:** Naoki Hosogaya, Takahiro Takazono, Rina Kawasaki, Hiroshi Yano.

**Formal analysis:** Naoki Hosogaya, Takahiro Takazono, Shinpei Morimoto.

**Methodology:** Naoki Hosogaya, Takahiro Takazono, Kenji Ota, Rieko Kiya, Yumi Shirai, Rina Kawasaki, Hiroshi Yano, Shinpei Morimoto, Rumiko Nakao, Yumiko Kanamaru, Yukari Yoshino, Yasuyuki Ishikawa, Chizu Fukushima, Hiroshi Yamamoto, Koichi Izumikawa, Katsunori Yanagihara, Hiroshi Mukae.

**Project administration:** Naoki Hosogaya, Takahiro Takazono, Rieko Kiya, Yumi Shirai.

**Writing – original draft:** Naoki Hosogaya, Takahiro Takazono.

**Writing – review & editing:** Hiroshi Yamamoto, Koichi Izumikawa, Katsunori Yanagihara, Hiroshi Mukae.
